# Vitamin K preserves gamma‐glutamyl carboxylase activity against carbamylations in uremia: Implications for vascular calcification and adjunct therapies

**DOI:** 10.1111/apha.70040

**Published:** 2025-04-09

**Authors:** Nadine Kaesler, Suresh Kaushik, Janina Frisch, Susanne Ziegler, Jochen Grommes, Alexander Gombert, Leticia Prates Roma, Christoph Kuppe, Joachim Jankowski, Jürgen Floege, Sofia de la Puente‐Secades, Rafael Kramann, Vera Jankowski

**Affiliations:** ^1^ Medical Clinic II University Hospital of the RWTH Aachen Aachen Germany; ^2^ Biosciences Cardiff University Cardiff UK; ^3^ Institute of Biophysics, Center of Human and Molecular Biology (ZHMB), Center for Gender‐Specific Biology and Medicine (CGBM) Saarland University Homburg Germany; ^4^ Marienhospital Aachen Clinic for Vascular Surgery Aachen Germany; ^5^ Clinic for Vascular Surgery University Hospital of the RWTH Aachen Aachen Germany; ^6^ Institute of Molecular Cardiovascular Research (IMCAR) University Hospital of the RWTH Aachen Aachen Germany; ^7^ Department of Pathology, Cardiovascular Research Institute Maastricht (CARIM) University of Maastricht Maastricht The Netherlands; ^8^ Aachen‐Maastricht Institute for CardioRenal Disease (AMICARE) University Hospital RWTH Aachen Aachen Germany

**Keywords:** carbamylation, chronic kidney disease, vascular calcification, vitamin K

## Abstract

**Aim:**

Vascular calcification contributes to morbidity and mortality in aging and is accelerated in diabetes and in chronic kidney disease. Matrix Gla Protein is a potent inhibitor of vascular calcification, which is activated by the vitamin K‐dependent gamma‐glutamyl carboxylase (GGCX). However, through a currently unidentified mechanism, the activity of GGCX is reduced in experimental uremia, thereby contributing to the promotion of vascular calcifications. In this study, we aim to identify the cause of these functional alterations and to stimulate the enzyme activity by potential GGCX binding compounds as a new avenue of therapy.

**Methods:**

Two rodent models of experimental uremia and human carotid plaques were assessed for GGCX activity and modifications, as well as calcification. In silico compound screening via BindScope identified potential binding partners of GGCX which were further validated in functional assays for enzymatic activity changes and for in vitro calcification. Mass spectrometry was applied to monitor molecular mass changes of the GGCX.

**Results:**

Mass spectrometry analysis revealed post‐translational modifications of the GGCX in uremic rats and mice, as well as in calcified human carotid plaques. Functional assays showed that the post‐translational carbamylation of GGCX reduced the enzyme activity, which was prevented by vitamin K2. Chrysin, identified by compound screening, stimulated GGCX activity, reduced calcium deposition in VSMCs, and oxidized GGCX at lysine 517.

**Conclusion:**

In conclusion, this study clearly demonstrates that the vitamin K‐dependent enzyme GGCX plays a significant role in uremic calcification and may be modulated to help prevent pathological changes.

## Graphical abstract

**Figure:**
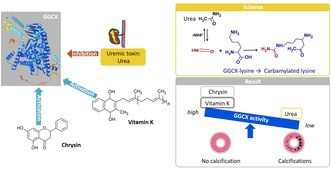
The source of the GGCX protein structure is uniprot, (image in the upper left of the graphical): this is the weblink: https://www.uniprot.org/uniprotkb/P38435/entry

## 1 INTRODUCTION

Vascular calcification is a serious complication in patients with cardiovascular disease (CVD) and chronic kidney disease (CKD), where calcium deposits build up in the blood vessel walls, leading to stiffness and impaired blood flow, contributing to the increased cardiovascular morbidity and mortality.^1^ While vascular calcification occurs with aging,^2^ it is markedly accelerated during specific pathologies such as diabetes^3^ and CKD^4^ due to the disruption or loss of mineral homeostasis, bone metabolism regulation, and calcification inhibitors. Vascular calcification is a highly complex process that is dynamically regulated by numerous stimulatory and inhibitory mediators, many of which are only partially understood. However, vascular calcification is a highly complex process that is actively regulated by numerous stimulators and inhibitors. One key player already identified in this multifactorially regulated process is the vitamin K‐dependent calcification inhibitor matrix Gla protein (MGP). MGP functions by inhibiting bone morphogenetic protein‐2 (BMP‐2)^5^ and directly binding to calcium phosphate crystals.^6^

MGP requires prior enzymatic activation by the vitamin K‐dependent gamma‐glutamyl carboxylase (GGCX) to exert its calcification inhibitory functions.^7^ A lack of functional MGP leads to stiffness and impaired blood flow, contributing to the increased cardiovascular morbidity and mortality.^8^

GGCX is an 88 kDa membrane‐bound enzyme, prone to post‐translational modifications that can influence its function, especially in the context of its role in mineralization and calcification inhibition. GGCX contains four functional domains: a carboxylase site, a propeptide binding site that docks the substrate, a propeptide binding site to stimulate the carboxylase and epoxidase activity, and a vitamin K binding site.^9^ Vitamin K epoxide is proposed to be located near the lysine at position 218^10^ and is further reduced stepwise in the vitamin K cycle to KH2. Several types of post‐translational modifications of GGCX have been reported, influencing its functional properties.^11^

Various mutations in the GGCX gene have been identified in humans and are associated with alterations in its enzymatic function. They manifest in a pseudoxanthoma elasticum‐like syndrome, including ophthalmological, dermal, cardiac, and osseous, as well as coagulation disorders, caused by calcifications of elastic fibers,^12^, ^13^ underscoring the critical role of GGCX in the regulation of calcification processes.

CKD is a major pathology that accelerates vascular calcifications and increases the risk of cardiovascular disease.^14^, ^15^ CKD patients exhibit a functional vitamin K deficiency, demonstrated by reduced circulating levels of activated vitamin K‐dependent proteins MGP.^16^ The levels of the inactive prothrombin precursor PIVKA (protein induced by vitamin K antagonist) are also increased in CKD compared to healthy controls.^17^ Therefore, this study aims to investigate the impact of CKD on GGCX and its downstream impact on vascular calcification.

We have already demonstrated, under experimental CKD conditions, reduced GGCX activity before severe vascular calcifications were present. This decrease in GGCX activity is reversible by vitamin K supplementation.^18^ Building on our previous findings, we aimed to investigate to elucidate the underlying causes for the decreased GGCX activity in CKD and identify potential intervention strategies to reduce pathological calcification. For this, we investigated whether post‐translational modifications are causal for the decreased GGCX activity in the context of CKD, as already shown for instance for sortilin, another calcification regulator.^16^ We investigated the effect of vitamin K2 supplementation in vivo and in vitro on the GGCX modifications. Moreover, a compound screening was performed to identify potential binding partners that could restore the reduced activity of GGCX and thus decrease VSMC calcification.

## 2 RESULTS

### 2.1 GGCX of murine CKD models was post‐translational modified

**FIGURE 1:**
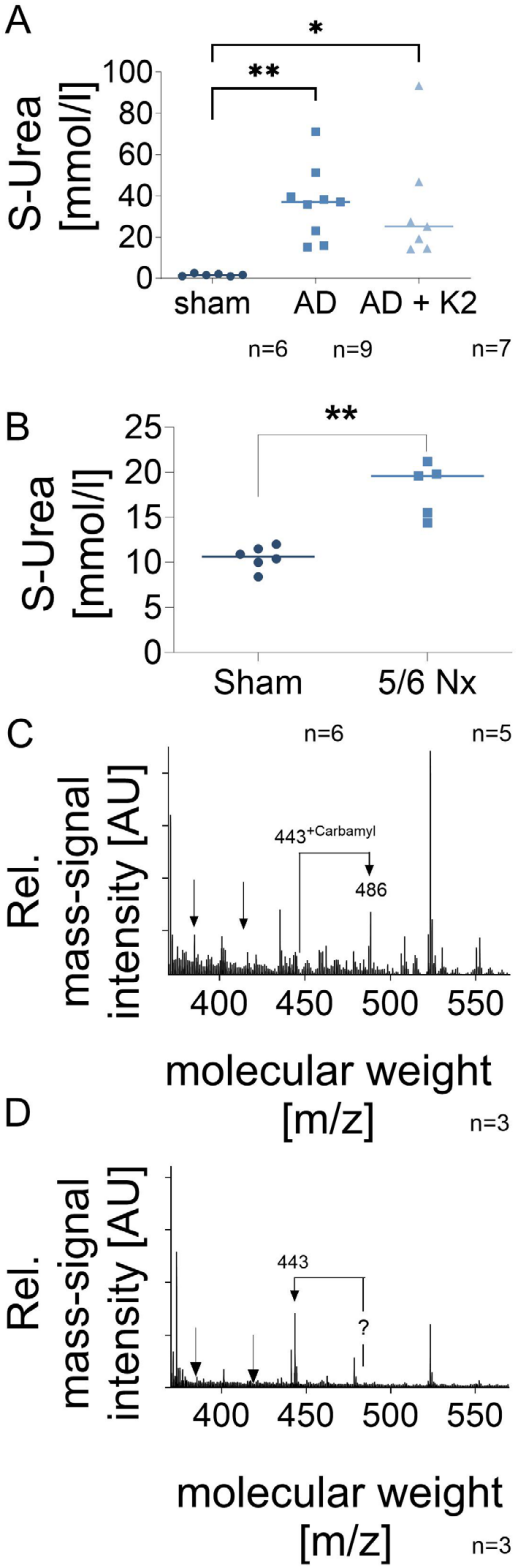
Samples were obtained from two established animal models of chronic kidney disease. These include Wistar rats with adenine‐induced nephropathy^18^ and C57BL/6 mice subjected to 5/6 nephrectomy in.^19^ (A) Serum urea in sham rats (*n* = 6), compared to serum urea in adenine nephropathy (AD) (*n* = 9), and serum urea in adenine nephropathy plus vitamin K2 (AD+K2) (*n* = 7; *p < 0.05; **p < 0.001). (B) Serum urea in sham‐operated mice (*n* = 6) compared to 5/6 nephrectomized mice (5/6Nx) (*n* = 5) (**p < 0.001). (C) Characteristic matrix‐assisted laser desorption/ionization (MALDI) MS spectrum of SD‐Gel pieces of aortas from adenine‐fed rats; the arrows indicate the modifications by carbamylation (shift of the molecular mass from 443 to 487) exemplary spectrum (*n* = 3). (D) Characteristic matrix‐assisted laser desorption/ionization (MALDI) MS‐spectrum of SDS‐Gel of aortas from adenine plus vitamin K2 fed rats with a distinct mass peak at position 443; exemplary spectrum (*n* = 3).

The serum urea concentrations of rats treated with adenine in the presence and absence of vitamin K2 increased significantly compared to the healthy control rats (Figure 1A). Adenine‐treated rats exhibited various soft tissue calcifications and increased serum phosphorous, as published earlier.^18^

Serum urea concentrations of mice after 5/6 Nx significantly increased from 10.53 ± 1.27 to 18.10 ± 2.96 mmol/L in 5/6 Nx mice compared to healthy controls (*N* = 5–6 *p* < 0.001) (Figure 1B). Arginines residues of GGCX, isolated from the liver of 5/6 Nx mice, were found to be carbamylated at amino acid positions 436, 672 (3 of 3 mice) and 480 (2 of 3 mice). No guanidinylations were detected by mass spectrometry in mice (Table 1). Spotty calcifications were visible in all aged mice; however, cbfa1 expression was significantly upregulated only in nephrectomized mice with an accordingly strong upregulation of FGF23, as previously reported.^19^

**TABLE 1:** Identified post‐translational carbamylations and guadinylations in adenine rats (kidney, pooled aorta) and 5/6 nephrectomized mice (liver), each to healthy controls, as well as in isolated rat microsomes (kidney) after in vitro carbamylation, to vehicle control, stated in ratio of numbers of identified samples with PTM to total number analyzed samples.

Species		Rat								Mouse	
Tissue		Kidney				Pooled aorta				Liver	
Group		In vitro	Healthy	Adenine 8 weeks	Adenine + Vit K	Healthy	Adenine 4 weeks	Adenine 8 weeks	Adenine +Vit K	Healthy	5/6 Nx
Guanidinylation	*GGCX Site*										
	Lysine 19	0/3	0/5	1/5	0/3	0/1	0/3	0/3	0/3	0/2	0/3
	Lysine 325	3/3	0/5	2/5	0/3	0/1	0/3	0/3	0/3	0/2	0/3
	Lysine 351	0/3	0/5	1/5	0/3	0/1	0/3	0/3	0/3	0/2	0/3
	Lysine 520	0/3	0/5	3/5	0/3	0/1	0/3	0/3	0/3	0/2	0/3
Carbamylation	*GGCX site*										
	Arginine 9	2/3	0/5	2/5	0/3	0/1	0/3	0/3	0/3	0/2	0/3
	Lysine 19	0/3	0/5	0/5	0/3	0/1	0/3	2/3	0/3	0/2	0/3
	Arginine 322	0/3	0/5	1/5	0/3	0/1	0/3	0/3	0/3	0/2	0/3
	Lysine 325	3/3	0/5	0/5	0/3	0/1	0/3	1/3	0/3	0/2	0/3
	Arginine 347	3/3	0/5	1/5	0/3	0/1	1/3	0/3	0/3	0/2	0/3
	Arginine 349	0/3	0/5	0/5	0/3	0/1	1/3	0/3	0/3	0/2	0/3
	Lysine 351	0/3	0/5	5/5	0/3	0/1	0/3	1/3	0/3	0/2	0/3
	Arginine 436	3/3	0/5	5/5	0/3	0/1	2/3	0/3	0/3	0/2	0/3
	Arginine 438	0/3	0/5	0/5	0/3	0/1	0/3	1/3	0/3	0/2	0/3
	Arginine 480	0/3	0/5	0/5	0/3	0/1	0/3	0/3	0/3	0/2	2/3
	Lysine 520	0/3	0/5	0/5	0/3	0/1	0/3	1/3	0/3	0/2	0/3
	Arginine 664	3/3	0/5	1/5	0/3	0/1	0/3	0/3	0/3	0/2	1/3
	Arginine 671	0/3	0/5	0/5	0/3	0/1	0/3	1/3	0/3	0/2	2/3
	Arginine 673	3/3	0/5	2/5	0/3	0/1	1/3	0/3	0/3	0/2	1/3
	Lysine 687	1/3	0/5	2/5	0/3	0/1	1/3	0/3	0/3	0/2	1/3
*x/y*	Number of samples with present modification/ total number of analyzed samples										

While GGCX, isolated from kidneys and aortas of healthy control rats, was not post‐translationally modified (Table 1), GGCX isolated from adenine‐fed rats was highly carbamylated, primarily at amino acid positions 9, 351, 436, 673, 687. Additionally, guanidinylation was detected at amino acid positions 19, 325, 351, 520 (Table 1, Figure 1C). GGCX isolated from the aortas of rats treated with both adenine and vitamin K2, was neither carbamylated nor guanidinylated (Table 1, Figure 1D). No significant correlation was observed between the relative extent of the modification and the GGCX activity (data not shown).

### 2.2 In vitro modifications mimic post‐translational modifications found in vivo

In vitro carbamylation of microsomal mixtures, isolated from healthy rat livers and kidneys, resulted in similar modification patterns to the ones found in GGCX in vivo isolated from adenine‐fed rats. Carbamylations occurred at arginines 19, 346, 436, 664, 673 and at lysines 325 and 687. Additionally, guanidinylation was present at lysine 325 (Table 1). In the presence of vitamin K2, no in vitro carbamylation was detectable by mass spectrometry (Table 1).

### 2.3 Human GGCX overexpressing cell line synthesized functional GGCX, which can be post‐translationally modified

**FIGURE 2:**
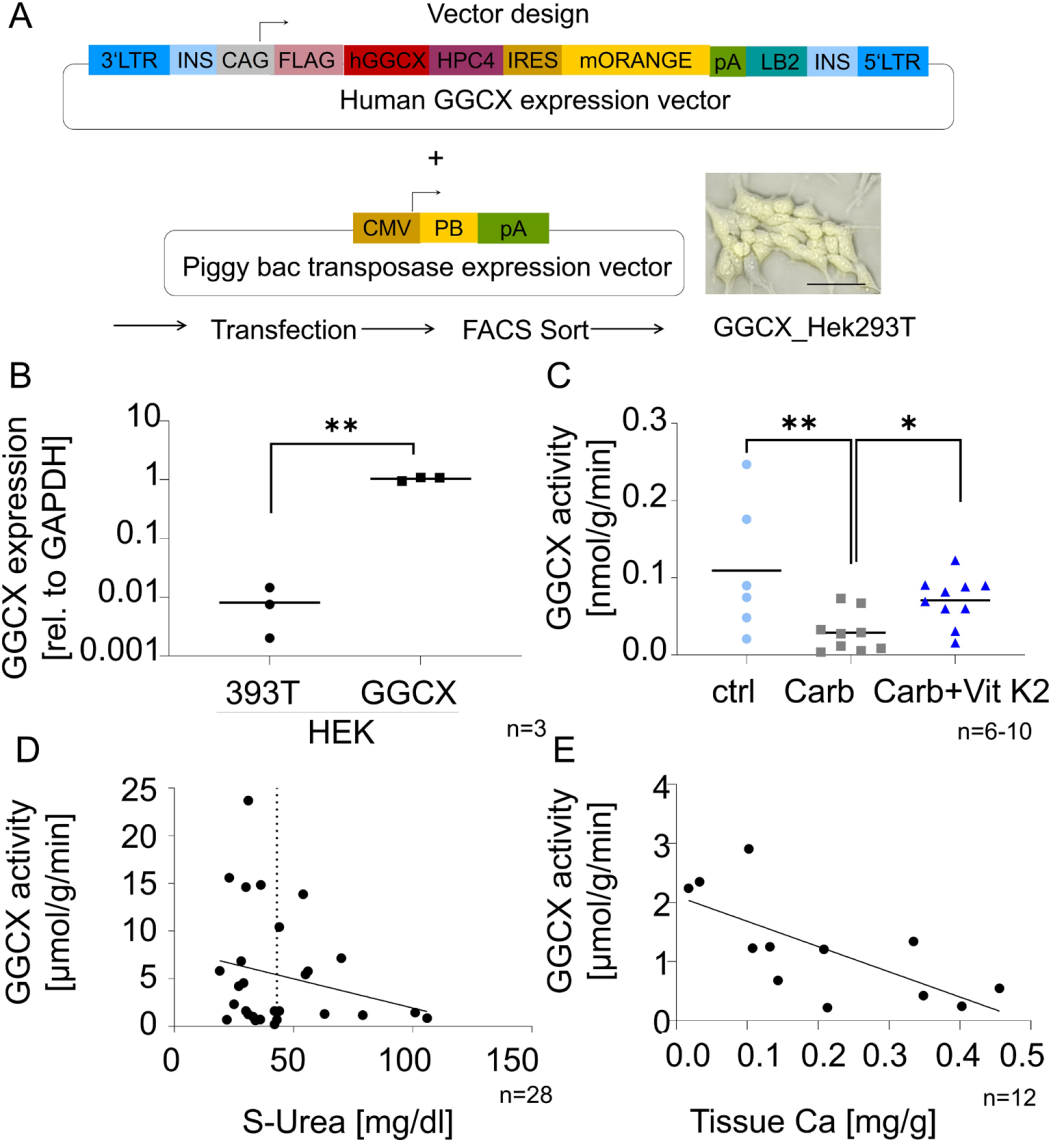
Overexpression and functional activity of human GGCX in HEK293T cells. Human GGCX was overexpressed in HEK239T cells, isolated by FACS sorting and purified membrane fractionation. The functionally active enzyme was used to assess the effects of carbamylation and vitamin K on GGCX function. (A) Human GGCX expression vector co‐delivered with Hyperactive PiggyBac transposase expression vector to obtain mOrange fluorescent GGCX overexpressing HEK293T cells; line = 90 µm. (B) Relative expression of GGCX mRNA in HEK293T cells compared to GGCX‐overexpressing HEK293T cells (**p<0.001). (C) In vitro carbamylation of purified GGCX: GGCX enzyme was purified by membrane fractionation from GGCX‐overexpressing HEK293T cells and subjected to in vitro carbamylation by potassium cyanate (carb), and in the presence of potassium cyanate plus vitamin K (carb +vitamin K), respectively (*p<0.05, **p<0.001). (D) GGCX activity in carotid arteries versus serum urea of carotid endarterectomy patients; linear regression not significant; dashed line to separate normal and increased serum urea levels. (E) GGCX activity versus total calcium content of renal arteries (*r* ^2^ = 0.50) (*p* = 0.01).

We generated a human GGCX overexpressing cell line as a validation tool based on human protein. Stable transfectants containing the GGCX‐mOrange overexpression transposon were enriched from 293T cells by FACS sorting for mOrange positive cells (Figure 2A). Transfection with the GGCX construct resulted in a 128‐fold increase in GGCX mRNA expression in HEK293T cells (*n* = 3) (Figure 2B).

Carbamylations were present after incubation with potassium cyanate at lysines 19, 43, and 687 as well as at arginine 438 (Table 2). No modifications were detected after treatment with vehicle control or after treatment with KOCN and vitamin K2 (Table 2).

**TABLE 2:** Identified post‐translational carbamylation and guanidinylation in human carotid artery samples (5 in total, 2 from dialysis patients) and in human GGCX from GGCX overexpressing HEK293T cells (each *n* = 3); stated in ratio of numbers of identified samples with PTM to total number of analyzed samples.

Species		Human				
Tissue		Carotid artery		GGCX_HEK293T		
Group		Plaque	Dialysis	Vehicle	KOCN	KOCN + K2
Guanidinylation	GGCX Site	No guanidinylations found				
Carbamylation	Lysine 19	2/2	2/2	0/3	3/3	0/3
	Lysine 91	2/3	2/2	0/3	3/3	0/3
	Arginine 438	0/3	2/2	0/3	3/3	0/3
	Lysine 578	3/3	2/2	0/3	0/3	0/3
	Lysine 687	3/3	0/2	0/3	3/3	0/3

### 2.4 In vitro modifications decreased human GGCX enzyme activity, restored by vitamin K2

In vitro carbamylation of the GGCX by KOCN resulted in a significant decrease in GGCX enzyme activity, which was prevented by adding vitamin K2 (Figure 2C) (*p* = 0.005 by *t*‐test to KOCN). No post‐translational modifications were detectable after incubations with KOCN plus vitamin K2 (Table 2).

To investigate the potential mechanism by which vitamin K protects from protein carbamylations, we incubated vitamin K1, vitamin K1 hydroquinone, and vitamin K1 epoxide each with urea. MS and MS/MS analyses revealed a carbamylation site on the vitamin K epoxide after incubation with urea (Figure S1).

### 2.5 Human arteries exhibited pathological alterations

To determine whether the alterations identified in rodent models are also relevant to human pathology, we analyzed human carotid and renal arteries for post‐translational modifications, GGCX activity, and calcification. 7% of the carotid endarterectomy patients were on Marcumar treatment, and 83% of the patients were male. The average age was 70 ± 9 years. CKD stages 2–4 were present in four patients. Von Kossa positive spots were observed in all carotid sections (Figure S2). Compared to non‐CKD patients (Figure S2‐1), the extent of von Kossa positive staining was higher in the presence of CKD (CKD stage 2 Figure S2‐2 and CKD stage 3 Figure S2‐3) and in Marcumar‐treated patients (Figure S2‐4). The extent of calcification ranged from 0.2 to 23.7 mg of total calcium per g tissue (Data not shown).

The GGCX activities of the carotid arteries ranged from 0.21 to 23.70 μmol/g/min. No significant correlations were found between GGCX and serum urea (Figure 2D) (*R* ^2^ = 0.05, *p* = 0.2) or estimated GFR (*R* ^2^ = 0.0002, *p* = 0.9) or carotid calcium content (*R* ^2^ = 0.01 *p* = 0.55). The data show a progressive decrease of GGCX activity with increasing urea concentration; however, this trend does not reach statistical significance in the present study due to the limited number of patients with pathologically elevated urea levels (Figure 2D). This limitation highlights the need for larger clinical studies to confirm this association. Furthermore, potential confounding factors such as metabolic and inflammatory changes in chronic kidney disease patients should be considered in future analyses to better understand the underlying mechanisms of GGCX regulation in this context.

GGCX isolated from carotid arteries (*n* = 5 out of 28, randomly selected) exhibited post‐translational carbamylation at lysine residues 19, 91, and 578. No guanidinylations were detectable (Table 2).

Nephrectomy patients were 75% male, and 50% of them were diagnosed with stage 3 CKD. Two patients had no CKD (16%), Stage 2 CKD was present in 25%, and one patient had stage 4 CKD (8%) (*n* = 12). Human renal arteries, derived from nephrectomized patients, were analyzed for total calcium and GGCX activity. On average, they had lower GGCX activities than the carotid arteries (Figure 2D,E). A negative correlation was found between GGCX activity and calcium content (*R* ^2^: 0.5, *p* = 0.01) (Figure 2E). Only arteries low in calcium exhibited high GGCX activity (Figure 2E).

### 2.6 Virtual compound screening identified chrysin as a potential GGCX binding partner

Next, we aimed to identify small molecules binding to the GGCX and altering the enzyme activity. Therefore, we initiated a virtual compound screening, providing binding probabilities of each compound.

To validate our screening model, we initially tested the binding prediction model by running hydroquinone, phylloquinone‐epoxide, and N‐ethylmaleimide (NEM), known as GGCX binding ligands on BindScope (www.playmolecules.org). The GGCX protein structure was downloaded from Alphafold.^20^ The predicted binding probabilities were 0.953 (hydroquinone), 0.9371 (phylloquinone‐epoxide), and 0.925 (NEM). For compound screening, 10 ligands were combined in one sdf file (Visualstudio, Microsoft) and prediction runs were performed via bindscope (two‐dimensional (2D) to three‐dimensional (3D) model). Potential ligands were randomly selected from various databases, including organic compounds, naturally occurring components, and their derivatives. In total, 675 ligands were initially screened for binding probability. Positive hits were further validated via Aceprep (3D to 3D model). The 2D to 3D conversion of the compounds was performed by using Aceprep (playmolecules.org.) .

Calindol, a calcimimetic, obtained a high binding probability of 0.96 in the 2D to 3D model, but failed in the 3D to 3D model, with a binding probability of 0 (Table 3). Furthermore, melatonin, initially identified through the 2D‐to‐3D screening, could not be confirmed using the 3D‐to‐3D (ligand‐to‐protein) model (Table 3). Following both models, a likely binding probability to the GGCX protein was computed for 5,7‐flavone (chrysin) and another calcimimetic, etelcalcetide (binding probabilities 0.86 and 0.56 for chrysin, 0.59 and 0.84 for etelcalcetide) (Table 3).

**TABLE 3:** Binding probabilities, identified by bindscope and KDEEP, of selected small molecules compounds.

	2D–3D Model	3D–3D Model
	Binding probability	
Chrysin	0.86	0.56
Etelcalcetid	0.59	0.84
Calcitriol	0.81	0.2
Calindol	0.96	0
Melatonin	0.83	0

The natural occurring bioflavone, chrysin, is described to mediate various potential health benefits.^21^ Chrysin promotes osteogenetic differentiation of osteoblasts^22^ and mediates vasorelaxive effects in aortic rings.^23^ Therefore, we focused on chrysin in the functional assays, GGCX activity, and VSMC calcification, in addition to etelcalcetide and calindol.

### 2.7 Chrysin modulated GGCX activity and VSMC calcification

**FIGURE 3:**
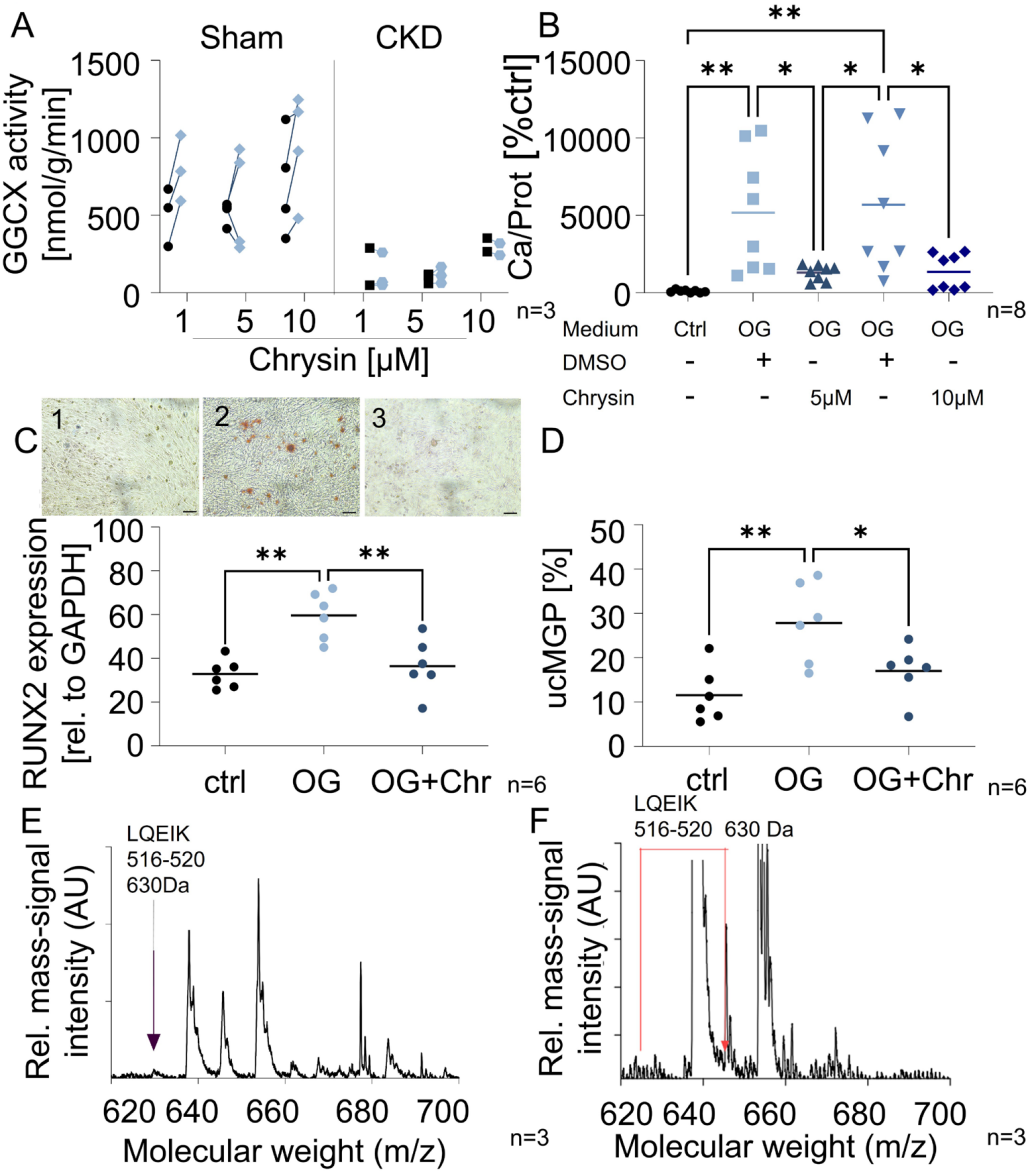
Effects of chrysin on GGCX activity and calcium content. Effects of chrysin were tested on GGCX containing microsomes isolated from livers from healthy and adenine‐fed rats (A, B, E) and on human VSMCs (C, D). (A) GGCX activity of liver microsomes, isolated from healthy and adenine‐fed rats, treated with chrysin (1, 5, 10 μM), each to vehicle control (0.001% DMSO) (*n* = 3). (B) Total calcium content in human immortalized VSMC, after 14 days of osteogenic treatment in the presence of chrysin (5, 10 μM), each to vehicle control (0.001% DMSO) and normal control medium (ctrl) (*n* = 8). (C) Real‐time PCR measurement, relative expression of RUNX2 to relative expression of GAPDH; ctrl: Normal control medium, OG: Osteogenic medium, OG + Chr: Osteogenic medium plus chrysin (10 μM); after 5 days of treatment; C1‐3 Exemplary images of alizarin red staining on VSMCs treated with (1) normal control medium, (2) osteogenic differentiation medium, (3) osteogenic differentiation medium plus chrysin (10 μM); scale bar =10 μM. (D) Flow cytometry analysis of ucMGP positive cells (% of single cells), 14 days after treatment with normal medium (ctrl), osteogenic medium (OG) and osteogenic medium plus chrysin (10 μM) (OG + Chr), respectively. (E) Characteristic mass spectrum of vehicle‐treated GGCX (intensity vs. mass to charge ratio) of fragments 516–520 (*n* = 3). (F) Characteristic mass spectrum of chrysin‐treated GGCX (intensity vs. mass to charge ratio) of fragments 516–520; shift in mass peak indicated by red arrow (*n* = 3).

The enzymatic activity of GGCX within isolated rat liver microsomes exhibited a notable decrease in rats with CKD compared to healthy controls (Figure 3A). Chrysin induced a mean 1.3‐fold increase in GGCX activity in microsomes isolated from healthy rats, but not from adenine rats (Figure 3A), failing to reach statistical significance among all groups here. The addition of chrysin to calcifying vascular smooth muscle cells (VSMC), induced by osteogenic medium, significantly decreased the total calcium deposition after 2 weeks of treatment (Figure 3B). Calcium deposits were visualized by alizarin red staining, highlighting several spots of positive staining only in the osteogenically treated VSMCs (Figure 3C–2), but not after treatment with normal control medium (Figure 3C–2) or osteogenic medium plus chrysin (Figure 3C–3). Osteogenic induction medium resulted in a significant upregulation of RUNX2 gene expression in VSMCs compared to cells cultured in normal control medium. The addition of chrysin to the osteogenic medium significantly reduced RUNX2 expression after 5 days of treatment (Figure 3C). Accordingly, osteogenic medium induced an increase in ucMGP compared to normal medium, which was significantly reduced by adding chrysin to the osteogenic medium, as shown by FACS analysis (Figure 3D).

Representative mass fingerprint spectra of tryptic digested SDS‐gel piece analyses revealed post‐translational oxidation at the peptide fragment LQEIK (516–520) after incubation with chrysin, whereas no oxidation was detected after incubation with vehicle control (0.001% DMSO) (Figure 3E,F). Figure S3 shows a representative MALDI TOF/TOF fragment mass spectrum of LQEIK with modification.

Both calcimimetics, etelcalcetide and calindol, exhibited no discernible effects on the activity of GGCX (Figure S4). The calcium content was significantly upregulated by osteogenic induction medium (Figure S4). Treatment with etelcalcetide significantly reduced the calcium content of VSMCs compared to the osteogenic control, whereas the treatment with calindol did not (Figure S4).

### 2.8 No impact on redox state by chrysin on calcifying VSMCs

As reactive oxygen species are described to be involved in vascular calcification processes^24^ and flavones, such as chrysin, are known to possess antioxidant activities,^25^ we aimed to investigate the role of the redox state in our VSMC calcification model and whether chrysin impacts it.

**FIGURE 4:**
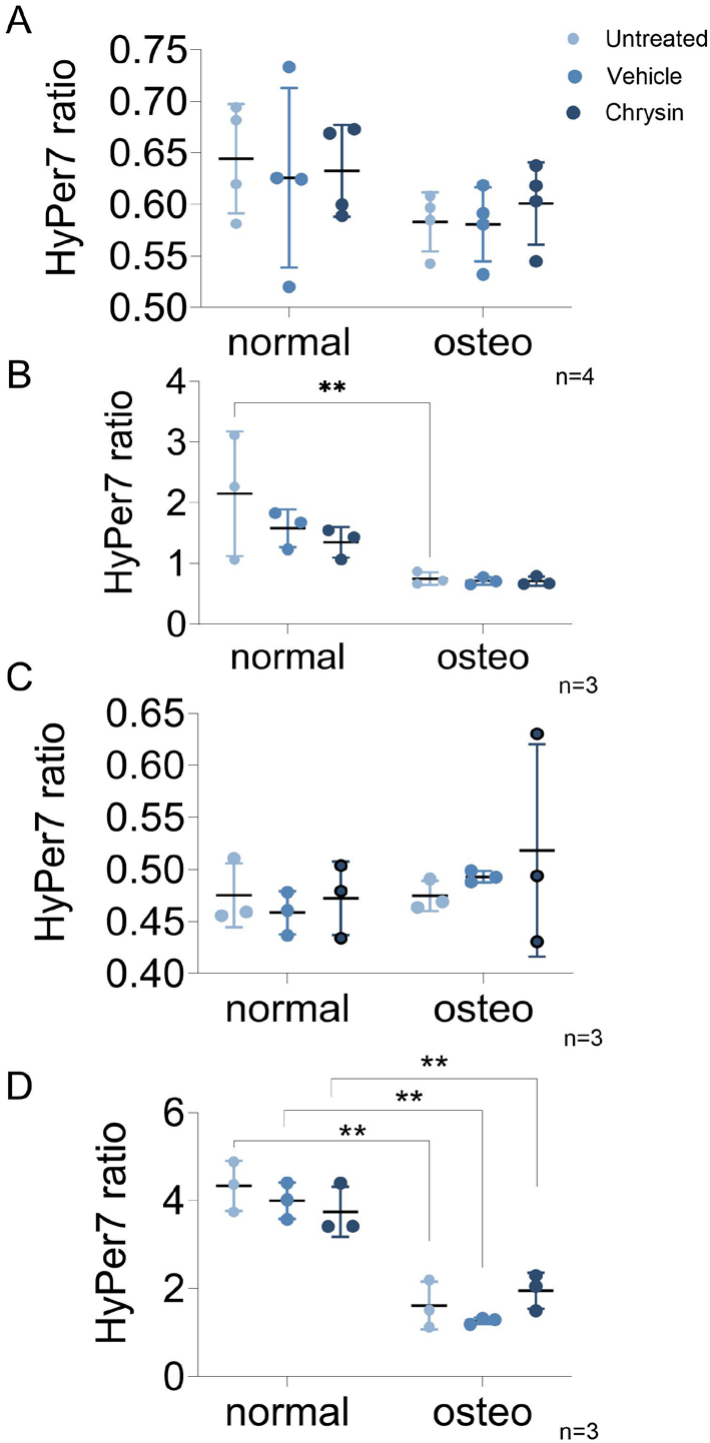
Redox status of VSMCs after 14 days of ostegenic differentiation, compared to normal medium, baseline and after stimulation with H_2_O_2_, all measured in measurement buffer. (A) Cytosolic baseline redox status of VSMCs treated with 5 μM chrysin plus osteogenic medium to the vehicle to standard medium as control condition; *n* = 3. (B) Cytosolic redox status after challenge with 10 μM H_2_O_2_, VSMCs treated with 5 μM chrysin plus osteogenic medium to the vehicle to standard medium as control condition (*n* = 3); (C) Mitochondrial baseline redox status of VSMCs treated with 5 μM chrysin plus osteogenic medium to the vehicle to standard medium as control condition (*n* = 4). (D) Mitochondrial redox status after challenge with 10 μM H_2_O_2_, VSMCs treated with 5 μM chrysin plus osteogenic medium to the vehicle to standard medium as control condition (*n* = 3).

VSMCs were cultivated in osteogenic induction medium in the presence of chrysin or vehicle, compared to standard and osteogenic medium. ROS levels were quantified using a reporter localized in the cytosolic or in the mitochondrial compartments of the VSMCs (Figure 4A–D). There were no differences in the baseline redox state in the cytosol (Figure 4A) or in the mitochondria (Figure 4C) after osteogenic differentiation. After treatment with H_2_O_2_, osteogenic differentiated cells responded significantly less than cells grown in physiologic medium conditions in the cytosol (Figure 4B) as well as in the mitochondria (Figure 4D). Chrysin failed to affect the redox state, both in the cytosol or in the mitochondria, of VSMCs (Figure 4A–D).

## 3 DISCUSSION

This study presents a novel approach to explain the decreased GGCX activity in uremia. Our findings provide data on post‐translational modifications across three different species and highlight their pathophysiological relevance in calcification processes. In addition, the study confirms previous findings of reduced GGCX activity in microsomes from adenine‐fed rats.^18^

Carbamylation, a non‐enzymatic post‐translational modification, results from the spontaneous reaction of isocyanic acid with free amino groups in proteins, peptides, and amino acids. This modification represents a pathologic mechanism that contributes to cardiovascular risk, protein dysfunction, inflammation, and disease progression. This process is highly relevant in CKD, mainly due to the accumulation of urea, which serves as a precursor for the formation of isocyanic acid.^26^ Carbamylation alters protein structure and function, like GGCX activity, and contributes to the progression of CKD and CKD complications, particularly cardiovascular disease. Carbamylation results from the interaction of cyanate, which occurs in equilibrium with isocyanic acid with lysine residues and the N‐terminal amino groups of proteins.

Under physiological conditions, cyanate levels are low; however, in CKD, the equilibrium is shifted toward increased cyanate formation because of increased urea concentrations, which in turn increases carbamylation. The resulting carbamylated biomolecules are strongly associated with CVD in CKD patients.^27^ The main biomolecules affected include albumin,^28^ hemoglobin, lipoproteins, and structural proteins such as collagen.^27^ Carbamylated low‐density lipoprotein (cLDL) is more readily taken up by macrophages, promoting foam cell formation and atherogenesis.^29^, ^30^ Additionally, carbamylation of fibrinogen and other coagulation factors can lead to thrombogenic complications.^31^, ^32^ Clinical studies have linked elevated carbamylated protein levels to increased mortality risk in CKD and end‐stage renal disease (ESRD) patients.^28^, ^33^

Here, we identified post‐translational modifications in CKD rats and mice, which were prevented by dietary supplementation with vitamin K2 in rats. By overexpressing human GGCX in HEK cells, we demonstrated that the carbamylation of the GGCX is a causal factor in the reduction in the enzyme activity.

Vitamin K, as a known cofactor and binding partner of the GGCX,^10^ might sterically prevent carbamylation or act as an acceptor for carbamylation itself. The identification of a carbamylation site at the vitamin K1 > O after incubation with urea provides support for the latter hypothesis.

In this study, we identified post‐translational modifications in calcified human carotid artery plaques and a correlation between calcium content and GGCX activity in renal arteries.

The study demonstrated a correlation between GGCX activity and urea concentration; however, this correlation did not reach statistical significance. This is likely due to the limited number of patients with pathophysiologically elevated urea concentrations within the studied cohort. Furthermore, the findings indicate that, in addition to urea concentrations, other factors influence GGCX activity, particularly in patients with physiological urea levels. Potential cofactors such as smoking and dietary habits should be considered in this context.^34^, ^35^ Given these observations, further studies are needed in patients with chronic kidney disease (stages 1–5D) to evaluate GGCX activity throughout the disease comprehensively. Special attention should be given to the impact of modifiable risk factors, including smoking and metabolic influences,^35^ to understand better their contribution to enzymatic regulation and potential therapeutic interventions.

Next, potential compounds interacting with the GGCX were additionally analyzed in the current study, aiming to increase the enzymatic activity further. Three independent bioassays yielded conclusive evidence for the efficacy of chrysin in preventing calcium deposition and stimulating GGCX activity.

First, chrysin was identified as a potential binding partner of the GGCX by virtual compound screening. Chrysin, a bioflavonoid present in passiflora, is an inhibitor of androgen to estrogen conversion enzyme aromatase^36^ and promotes cancer cell apoptosis^37^ as well as immunogenic cell death of cancer cells.^38^ Further, the potential benefits of chrysin are currently under investigation, such as its anti‐inflammatory, cardio‐protective, neuro‐protective, and lipid‐lowering effects.^21^

In the current study, chrysin induced an average increase of 1.3‐fold in GGCX activity in GGCX overexpressing HEK cells. Evidence for a direct interaction between chrysin and GGCX is supported by the detection of an oxidation at lysine 520, observed exclusively after incubation with chrysin. The calcium deposition in in vitro calcifying VSMCs by chrysin was remarkably reduced, with about one‐fourth of the amount in vehicle‐treated cells, as confirmed by alizarin red staining and early RUNX2 gene expression. Moreover, chrysin reduced ucMGP levels, indicating a stimulatory effect on vitamin K‐dependent gamma carboxylation. While these findings suggest a direct role of chrysin in enhancing GGCX activity, other potential mechanisms playing a role besides GGCX stimulation still cannot be excluded. Matrix metalloproteinases, such as MMPs 2 and 9, can initiate uremic vascular calcifications^39^ and inhibition of MMP9 expression was observed after chrysin pre‐treatment in gastric cancer cells.^40^ Flavones, such as chrysin, are known antioxidants. However, the antioxidant properties of chrysin do not seem to be responsible for the reduced calcium deposition in VSMCs. Due to the unpublished composition of the applied commercial osteogenic medium, we cannot evaluate any potential impact of containing antioxidative compounds, such as ascorbic acid, which is commonly used in combination with beta‐glycerophosphate to stimulate calcification of cell cultures. However, any spillover effects seem unlikely as the redox measurements are performed in PBS buffer.

Microsomal GGCX, isolated from uremic rats, was not stimulated by chrysin. Interestingly, the oxidation site identified by MS and MS/MS is located at one of the fragments that showed carbamylations (Lysine 520) in the presence of uremia. Assuming a binding of chrysin to the GGCX at this position could explain why the GGCX isolated from CKD rats was not stimulated by the addition of chrysin. The current carbamylation occurring at the GGCX might serve as an endogenous inhibitor, impeding the chemical binding of chrysin to the GGCX. Further experiments should investigate the effect of chrysin on calcifying VSMCs containing a carbamylated GGCX and the effect of chrysin in CKD models, with and without vitamin K, and which pathologies might benefit from chrysin supplementation.

Second, the calcimetics calindol and etelcalcetide, identified by virtual compound screening, did not change the GGCX activity. However, etelcalcetide reduced the calcium content of calcifying VSMCs, presumably due to its interaction with the calcium‐sensing receptor, as described in a rat model of renal insufficiency,^41^ rather than through an interaction with the GGCX.

## 4 METHODS

### 4.1 Animal models

Five Wistar rats per group were fed a control diet or a 0.75% adenine diet over 7 weeks with an adenine‐free interphase of 1 week, as described previously.^18^ The initial body weight was 300–400 g. Vitamin K2 (Menaquinone 4, Sigma Aldrich, USA) was added to the adenine diet (Altromin, Germany) at a dosage of 500 mg/kg chow.

In addition, 35‐ to 38‐week‐old female mice C57BL/6 underwent 5/6 nephrectomy (Nx). The surgery was performed using 2.5% isoflurane anesthesia on a 37°C warming plate. After the flank incision, the left kidney mass was reduced to one‐third. The contralateral kidney was removed entirely. After 1 week of recovery, a high phosphate diet was initiated (1.65% phosphorous, AB diets, The Netherlands). Mice were killed 12 weeks later. Samples were snap‐frozen and stored at −80°C until analysis.^19^ Plasma and liver samples from previously published animal groups were included in the study, as well.^18^, ^19^ The animal experiments were approved by the local committee (LANUV 84‐02.04.2011.A144 and 84‐02.04.2016.A1509).

### 4.2 Serum biochemistry

Serum urea and serum creatinine in rats and mice were measured by clinical routine methods (Vitros 350, USA).

### 4.3 SDS–PAGE electrophoresis

Membranous protein fractions were separated by 10% SDS–polyacrylamide gel electrophoresis (PAGE) for 45 min at 100 V/20 mA (BioRad, Germany). Mouse livers were separated by SDS–Page after homogenization in RIPA buffer (1% NP40), 0.5% sodium‐desoxycholate, 0.1% SDS, EDTA‐free protease inhibitor cocktail (Roche, Switzerland). After electrophoresis, the band for the GGCX at 88 kDa, identified by a reference, was manually cut out and analyzed at 88 kDa for mass spectroscopic analyses.

### 4.4 MALDI TOF/TOF‐mass spectrometry

Five supernatants of samples from the enzyme‐rich microsomes were collected for the mass spectrometric analysis, as previously described.^18^ For mass spectrometry analysis, SDS electrophoresis was initially performed. The SDS gel band containing GGCX was digested by trypsin and analyzed by matrix‐assisted laser desorption/ionization time of flight mass spectrometry (MALDI TOF/TOF‐mass spectrometry) using MS and MS/MS techniques (Ultraflex and Rapiflex, Bruker Daltronik, Germany). Next MS/MS fragments were analyzed using the Lift‐option of the MALDI‐TOF/TOF mass spectrometer. Calibrated and annotated spectra were subjected to the database search Swiss‐Prot (http://www.expasy.org/) using the software tool “Bruker Bio‐Tool 3.2” and the “Mascot 2.2 search engine” (Matrix Science Ltd., London, UK) for protein identification.

### 4.5 In vitro carbamylation

In vitro carbamylations were performed using microsomes isolated from healthy Wistar rat livers and kidneys, as well as membrane proteins isolated from GGCX‐HEK293T and HEK293T cells. For carbamylation, the samples were incubated for 1 h with potassium cyanate (Sigma Aldrich, USA) (KOCN, 2 mM in PBS), at room temperature. Carbamylated GGCX was co‐incubated with vitamin K2 (Menaquinone‐4, 10 μg/mL) or vehicle, 30 min prior to the GGCX activity measurement.

Vitamin K1 (Sigma Aldrich, USA) was reduced by incubation with dithiothreitol (20 mM, AppliChem, Germany) for 36 h at 37°C, protected from light. For oxidation, vitamin K was epoxidized by H_2_O_2_ (5%, AppliChem, Germany) for 12 h at 37°C, protected from light. The purity of both compounds was controlled by reversed‐phase HPLC, as published.^18^

### 4.6 GGCX expression vector construction

Human GGCX cDNA was synthesized as a gBlocks HiFi Gene Fragment from IDT. The gBlock fragment incorporated a FLAG tag at the N‐terminus to facilitate detection by western blotting and a C‐terminus HPC4 tag to facilitate protein purification^42^ The gBlock fragment was cloned into a PiggyBac transposon expression vector by restriction‐free cloning using NEB HiFi DNA assembly (New England Biolabs, USA). The PiggyBac transposon vector backbone (kind gift from Dr. Keisuke Kaji) incorporated Chicken B‐globin insulators 5′ and 3′ of GGCX and a laminin early replicator fragment to mitigate transgene silencing.^43^ The constitutive synthetic promoter CAG drives GGCX expression, and the vector also incorporated the fluorescent reporter mOrange to select GGCX‐expressing cells.

### 4.7 GGCX expressing HEK293T cell line

The GGCX PiggyBac transposon expression vector (2 μg) along with 500 ng of “Hyperactive PiggyBac transposase” expression vector (Kind gift of Dr. Yosuke Yusa) was delivered to HEK293T cells plated in a well of a six‐well plate using Lipofectamine LTX plus (Thermo Fisher, USA) as per the manufacturer's protocol. Forty‐eight hours after transfection, the cells were expanded and subcultured for a week, after which the cells were sorted on a Sony SH800 cell sorter for mOrange expression to select GGCX‐expressing cells. Primers for human GGCX sequencing were F1: GATCTTCATTGTGTACTTCATTGC, F2: CACAGAGTCGGCGATGGA, F3: CCATGAGCGATTCTTCCG.

### 4.8 Isolation of membrane proteins

Enzyme‐rich microsomes from livers, kidneys, and aortas from diseased and healthy mice, rats, or human vessels were isolated by ultracentrifugation, as described previously.^44^ Murine aortas were pooled before the isolation of microsomes (each single pool consisted of *n* = 3 aortas).

Membrane proteins were isolated from the GGCX overexpressing HEK293T cells and from HEK293T cells with the MEM‐PerTM Plus Membrane Protein Extraction Kit (Thermo Fisher, USA) in the presence of phosphatase (PhosSTOP, Merck Millipore, Germany) and protease inhibitors (Complete Mini, EDTA free, Switzerland). The isolated membrane protein fractions were stored at −80°C.

### 4.9 GGCX activity assay

The GGCX activity assay was performed using microsomes derived from human carotid arteries and human renal arteries, as well as isolated human membrane proteins from HEK293T and GGCX overexpressing HEK293T cells. To generate an enzyme pool for compound analysis, microsomes isolated from rat livers (healthy or CKD) were pooled. A synthetic, fluorescence‐labeled hexapeptide FLEFLK‐FITC (0.5 mM, purity >90%, Biomatik, Canada) was incubated with 250 μg of membrane proteins, reduced vitamin K1 (K1H_2_, 100 μg/mL), 5 mM DTT, 10 mM MnCl_2_, and 0.5% (w/v) 3‐[(3‐cholamidopropyl) dimethylammonio]‐1‐propane sulfonate. After 30 min, the reaction was stopped by adding 2 times volumes of ice‐cold methanol.^44^ Microsomes from rat livers were incubated with chrysin (1, 5, 10 μM in 0.001% aqueous DMSO, Sigma Aldrich, USA) or etelcalcetide (100 μM, in H_2_O, Parsabiv®) or calindol (1–10 μM in 0.001% aqueous DMSO, Biozol, Germany), each to vehicle controls (0.001% DMSO or H_2_O), 30 min prior to the GGCX activity assay. GGCX activity was calculated as nmol/g protein/min.

### 4.10 Tissue and cellular calcium

The total calcium content in tissues and deposits within VSMCs were quantified using the o‐cresolphthalein method (Randox, UK). Total protein was measured using the bicinchoninic acid (BCA) method (Thermo Fisher, USA). The calcium content of tissues was normalized to tissue wet weights. The calcium content of VSMC was normalized, for each replicate, to total protein content and baseline calcification of the control cells (% of control). The calcium phosphate deposits in vessels were stained by von Kossa staining.^18^

Calcium deposition in VSMCs was additionally visualized by alizarin red staining. Therefore, paraformaldehyde‐fixed VSMCs were stained for 30 min at room temperature, protected from light, with Alizarin red solution (Millipore, USA) and imaged using a Leica DMI8 microscope (Leica, Germany).

### 4.11 Patients samples

We collected 28 carotid arteries of patients undergoing endarterectomy surgery for carotid stenosis. The samples were snap‐frozen and stored at −80°C until analysis. Enzyme‐rich microsomes were assayed for GGCX activity and total calcium content. Additionally, a subset of five samples was analyzed for post‐translational modifications by MALDI TOF/TOF‐mass spectrometry.^45^

Normal human kidney arteries were obtained from patients undergoing nephrectomy for localized cancer, and were histologically verified. The arteries were processed as described previously.^46^

The glomerular filtration rate (GFR) was calculated using the CKD EPI 2009 equation.^5^, ^47^

The local ethical committee approved all human tissue protocols (EK 108/22 and EK‐016/17).

### 4.12 Virtual compound screening

To virtually screen for potential binding partners of the GGCX, we used the platform “*playmolecules.com*.” A 3D structure of the GGCX structure was downloaded from Alphafold. For a screening of 2D ligands, we used the protein‐ligand binding predictor Bindscope (https://playmolecule.com/BindScope/). For 3D‐to‐3D screening, we used KDEEP (KDEEP: protein‐ligands absolute binding affinity prediction via 3D neural networks 19). SDF files were downloaded from various platforms (Zinc, ARONIS, EXIMED, CHemBl). For KDEEP, 2D SDF files were converted into 3D by Aceprep (https://www.playmolecule.com/aceprep/). The binding score ranges from 0 to 1.

### 4.13 Cell cultures

Human vascular smooth muscle cells (hVSMC)^45^, ^48^ were cultivated in DMEM (Gibco, Glutamax 4,5 g/ glucose) with 10% FCS (10270‐106, Gibco, USA) and 1% penicillin–streptomycin (Thermo Fisher, USA). Osteogenic differentiation was done for 14 days (StemXVivo) osteogenic medium (Bio‐Techne, USA) compared to the normal growth medium (41 965 039, Gibco, USA). Chrysin (1, 5, 10 μM) or etelcalcetide (100 μM) (Parsabiv®), each to vehicle controls, were supplemented to differentiation or growth medium, simultaneously.

HEK293T cells and GGCX overexpressing HEK293T cells were cultivated in DMEM (low glucose, Gibco, USA) supplemented with 10% (Gold Plus, Bio&Sell, Germany) and 1% penicillin–streptomycin (Thermo Fisher, USA).

### 4.14 RT‐PCR in VSMCs

After 5 days of treatment, hVSMCs were resuspended in RLT buffer containing 1% beta mercaptoethanol (Merck, Germany) and stored at −70°C until further analysis. RNA isolation was performed with the Qiagen RNeasy mini Kit (Qiagen, Germany), according to the manufacturer's instructions. RNA was quantified on a nanodrop (Thermo Fisher, USA). The cDNA was synthesized with a random primer (11034731, Roche, Switzerland) in first strand buffer (28025–0113, Invitrogen, USA) and in the presence of a RNase inhibitor (Promega, USA). RT‐PCR was performed by the qPCR Core kit with SYBR green (Eurogentec, Belgium) on a Bio‐Rad CFX OPUS 96 system (Bio‐Rad, USA).

RT‐PCR primers were GAPDH fwd AGCCACATCGCTCAGACACC, GAPDH rev GCGCCCAATACGACCAAAA; Acta2 fwd ACTGCCTTGGTGTGTGACAA, Acta2 rev CACCATCACCCCCTGATGTC RUNX2 fwd CCGCCTCAGTGATTTAGGGC, RUNX2 rev GGGTCTGTAATCTGACTCTGTCC.

### 4.15 Flow cytometry

After 14 days of treatment, cells were harvested, fixed, and permeabilized using “Fix & PERM” buffer (Thermo Fisher, USA). HVSMCs were stained with an uncarboxylated (uc) MGP antibody (Kind gift from Leon Schurgers).^48^ Staining intensity was quantified by gating on single cells against an unstained control, on a BD FACS Fortessa and analyzed by “FACS Diva software”, and then the percentage of positively stained cells was calculated.

### 4.16 H_2_O_2_ measurements

HyPer7 plasmids^49^ with corresponding targeting sequences for mitochondrial and cytosolic expression were purchased from Addgene (136 470 and 136 467, Addgene, USA) in pCS2 backbones. pLVX vector backbone was kindly provided by Tobias Dick (DKFZ Heidelberg). Cloning was performed according to standard procedures. Lentiviral particles were generated through Ca_2_PO_4_ transfection of HEK293FT cells. Released lentiviral particles were collected 48 h after transfection and concentrated via Amicon‐15‐Ultra centrifugal filter columns (100 kDa MWCO). Immortalized hVSMC^48^ were transduced 1:3 with lentivirus in the presence of 8 μg/mL polybrene and selected for transgenic cells with puromycin for 14 days.

Measurements were performed in measurement buffer (PBS with Ca^2+^ and Mg^2+^, 4.5 g/L glucose) in an Axio Observer 7 imaging system (Zeiss, Germany) equipped with a mercury arc lamp (HXP 120 V) was used for sensor imaging. Filter sets and beam splitters predefined for excitation were either set at 405 (BP 430/60, FT 500) or 488 nm (BP 470/40 HE, FT 496 HE), and emission was detected at 500 to 550 nm. The recording was performed using the ZEN 2.6. Software (Zeiss, Germany). Measurements were taken in 20‐second intervals over a total of 40 min on a temperature‐controlled measurement unit at 37°C. For the first 20 min, the basal redox state was recorded while continuously perfusing with measurement buffer at 1 mL/min using a peristaltic pumping system. At 20 min, a buffer was changed to 10 μM H_2_O_2_ for another 10 min. HyPer7 ratios were calculated as fluorescence ratios (488/405 nm). Per replicate, approximately 30 single cells were selected as ROIs and analyzed individually for their HyPer7 ratios throughout the entire measurements. Means were calculated from baseline redox state and low‐dose H_2_O_2_ challenge. Each experiment was performed in triplicate in independent experiments using cells from different passages. Every replicate was further summarized to obtain one single data‐point (means from 30 cells) per condition.

### 4.17 Statistics

The Shapiro–Wilk normality test and visual classification were performed to evaluate whether the data followed a Gaussian distribution. Student's *t*‐test with independent variance was applied to check for differences between two groups for parametric data. The significance level was set to *p* < 0.05 (*). Data are mean ±SD unless stated otherwise. Simple linear regressions were performed to analyze the relationship between GGCX activities in human arteries and calcium content, GFR and urea. Statistics of redox experiments included two‐way ANOVA with Sidak's post‐test for multiple comparisons without matching values. Final data are presented as means from ≥3 individual experiments ±SD. The software used were GraphPad Prism 10 and IBM statistics SPSS 29.

## 5 CONCLUSION

In conclusion, this study demonstrates that in uremia, a high vitamin K intake is required to maintain the physiological function of the GGCX. Vitamin K protects GGCX from carbamylations and prevents a decline in its activity both in vivo and in vitro. Since post‐translational modifications were detected in endarterectomy specimens of non‐CKD patients as well, further studies should evaluate the impact of vitamin K supplementation in atherosclerotic lesions. Exploring additional treatments aiming at optimizing vitamin K metabolism, such as chrysin, holds promise in mitigating non‐uremic calcification or as an adjunct therapeutic intervention in addition to vitamin K supplementation.

## AUTHOR CONTRIBUTIONS

**Nadine Kaesler:** Conceptualization; investigation; funding acquisition; writing – original draft; methodology; validation; visualization; writing – review and editing; formal analysis. **Suresh Kaushik:** Investigation; methodology; writing – review and editing; writing – original draft; formal analysis. **Janina Frisch:** Investigation; writing – original draft; writing – review and editing; visualization; formal analysis. **Susanne Ziegler:** Investigation; formal analysis; methodology; writing – review and editing. **Jochen Grommes:** Methodology; writing – review and editing; validation. **Alexander Gombert:** Methodology; writing – review and editing; validation. **Leticia Prates Roma:** Formal analysis; methodology; writing – review and editing. **Christoph Kuppe:** Writing – review and editing; formal analysis; validation. **Joachim Jankowski:** Conceptualization; funding acquisition; writing – original draft; writing – review and editing; visualization; validation; project administration; supervision; resources. **Jürgen Floege:** Conceptualization; funding acquisition; writing – original draft; writing – review and editing; formal analysis; supervision; resources. **Sofia de la Puente‐Secades:** Investigation; writing – review and editing. **Rafael Kramann:** Conceptualization; funding acquisition; writing – original draft; writing – review and editing; project administration; supervision; resources. **Vera Jankowski:** Investigation; funding acquisition; writing – original draft; writing – review and editing; visualization; formal analysis; validation.

## FUNDING INFORMATION

VJankowski is funded by the Deutsche Forschungsgemeinschaft (DFG, German Research Foundation) by the Transregional Collaborative Research Centre (TRR 219; Project‐ID 322900939), (subproject S‐03; NK and RF subproject C‐01), (INST 948/4S‐1); CRU 5011 project number 445703531, Cost‐Action CA 21165, IZKF Multiorgan complexity in Friedreich Ataxia, and Phase Transition in Disease (1‐1), ERA‐PerMed (ERA‐PERMED2022‐202‐KidneySign).

## CONFLICT OF INTEREST STATEMENT

All authors declare no conflict of interest related to this work.

## Supporting information


**Figure S1:** Representative mass spectra of vitamin K1, incubated with and without urea (2 μM); vitamin K1 (A), vitamin K1 with urea (B), reduced vitamin K (KH2) (C). K1H2 with urea (D), vitamin K1 epoxide (Vit K ox) (E), vit K ox with urea (F).
**Figure S2:** 1–4 Exemplary von Kossa stainings of human carotid arteries from carotid endarterectomy surgery, (1) non‐CKD patient, (2) stage 2 CKD patient, (3) stage 3 CKD patient, (4) no CKD, Marcumar receiving patient; all images 100x.
**Figure S3:** Characteristic fragment mass spectrum of chrysin‐treated GGCX showing an oxidation at the molecular mass 658da (LQ*EIK).
**Figure S4:** GGCX activity and calcium deposition after treatment with calcimimetics, which were suggested for potential GGCX binding via virtual compound screening; (A) GGCX activity in healthy rat liver microsomes after incubation with calindol (Cal), or etelcalcetide (Etel) to vehicle control (Ctrl); (B) calcium deposition in VSMCs after incubation with calindol or etelcalcetide, both in combination with osteogenic medium (OG), compared to normal medium control (Ctrl) and to osteogenic medium alone.

## Data Availability

The data that support the findings of this study are available from the corresponding author upon reasonable request.
