# Supplementary material for: Vitamin K preserves gamma‐glutamyl carboxylase activity against carbamylations in uremia: Implications for vascular calcification and adjunct therapies
Source: Acta Physiol (Oxf). 2025 Apr 9;241(5):e70040. doi: 10.1111/apha.70040 (PMC11979876; doi:10.1111/apha.70040)
Supplement: Supplementary file 1 — Figure S1: Representative mass spectra of vitamin K1, incubated with and without urea (2 μM); vitamin K1 (A), vitamin K1 with urea (B), reduced vitamin K (KH2) (C). K1H2 with urea (D), vitamin K1 epoxide (Vit K ox) (E), vit K ox with urea (F). Figure S2: 1–4 Exemplary von Kossa stainings of human carotid arteries from carotid endarterectomy surgery, (1) non‐CKD patient, (2) stage 2 CKD patient, (3) stage 3 CKD patient, (4) no CKD, Marcumar receiving patient; all images 100x. Figure S3: Characteristic fragment mass spectrum of chrysin‐treated GGCX showing an oxidation at the molecular mass 658da (LQ*EIK). Figure S4: GGCX activity and calcium deposition after treatment with calcimimetics, which were suggested for potential GGCX binding via virtual compound screening; (A) GGCX activity in healthy rat liver microsomes after incubation with calindol (Cal), or etelcalcetide (Etel) to vehicle control (Ctrl); (B) calcium deposition in VSMCs after incubation with calindol or etelcalcetide, both in combination with osteogenic medium (OG), compared to normal medium control (Ctrl) and to osteogenic medium alone. [file APHA-241-e70040-s001.docx]

Vitamin K preserves gamma glutamyl carboxylase activity against carbamylations in uremia: Implications for vascular calcification and adjunct therapies

Nadine Kaesler^1*^, Suresh Kaushik^2^, Janina Frisch^3^, Susanne Ziegler^1^, Jochen Grommes^4^, Alexander Gombert^5^, Leticia Prates Roma^3^, Christoph Kuppe^1^, Angel Argiles^6^, Joachim Jankowski^7,8,9^, Jürgen Floege^1^, Sofia de la Puente-Secades^7^, Rafael Kramann^1ⴕ^, Vera Jankowski^7ⴕ^

1: University Hospital of the RWTH Aachen, Medical Clinic II, Aachen, Germany

2: Biosciences, Cardiff University, Cardiff, United Kingdom;

3: Institute of Biophysics, Center of Human and Molecular Biology (ZHMB), Center for Gender-specific Biology and Medicine (CGBM), Saarland University, Homburg, Germany;

4: Marienhospital Aachen, Clinic for vascular surgery, Aachen, Germany

5: University Hospital of the RWTH Aachen, Clinic for vascular Surgery, Aachen, Germany

6: Université de Montpellier, Montepellier, France

7: University Hospital of the RWTH Aachen, Institute of molecular cardiovascular research (IMCAR), Aachen Germany

8: Department of Pathology, Cardiovascular Research Institute Maastricht (CARIM), University of Maastricht, The Netherlands

9: Aachen-Maastricht Institute for CardioRenal Disease (AMICARE), University Hospital RWTH Aachen, Aachen, Germany


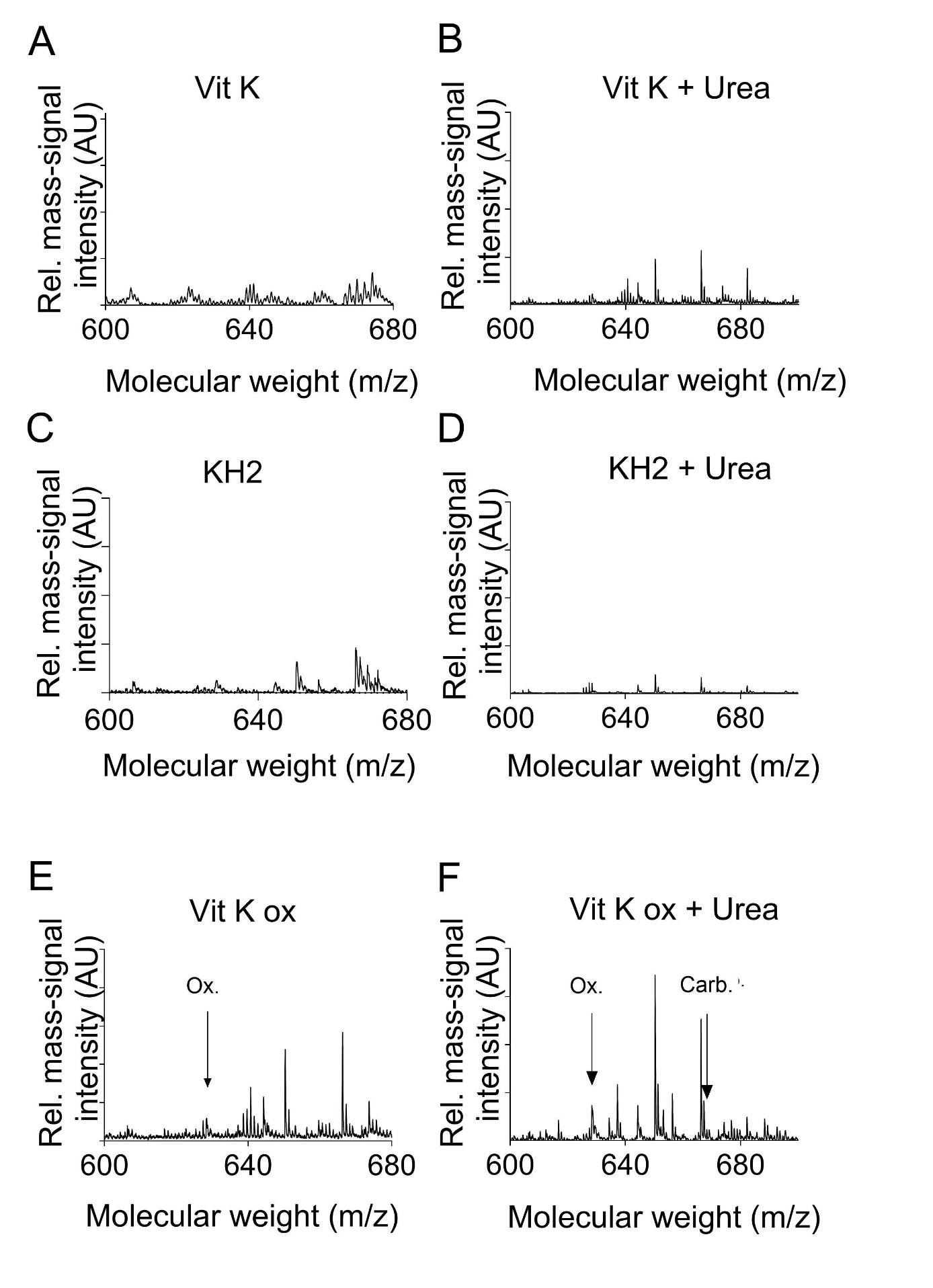


**Figure S1**: Representative mass spectra of vitamin K1, incubated with and without urea (2µM); Vitamin K1 (A) Vitamin K1 with urea (B), reduced vitamin K (KH2) (C). K1H2 with urea (D), Vitamin K1 epoxide (Vit K ox) (E), Vit K ox with urea (F)


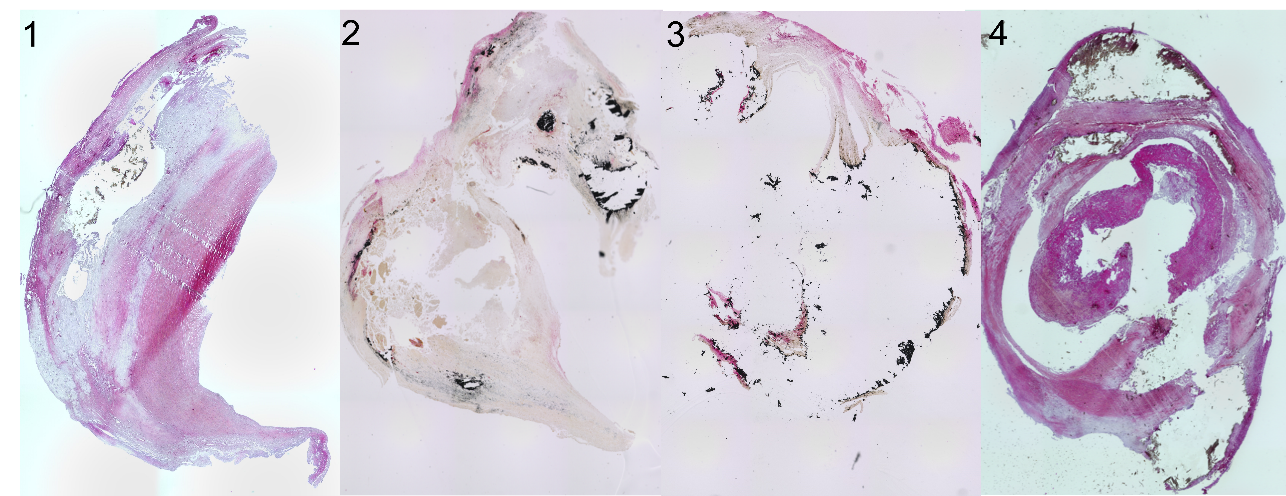


**Figure S2**: 1-4 Exemplary von Kossa stainings of human carotid arteries from carotid endarterectomy suregery (1) non CKD patient (2) stage 2 CKD patient (3) stage 3 CKD patient (4) no CKD, marcumar receiving patient; all images 100x, merged


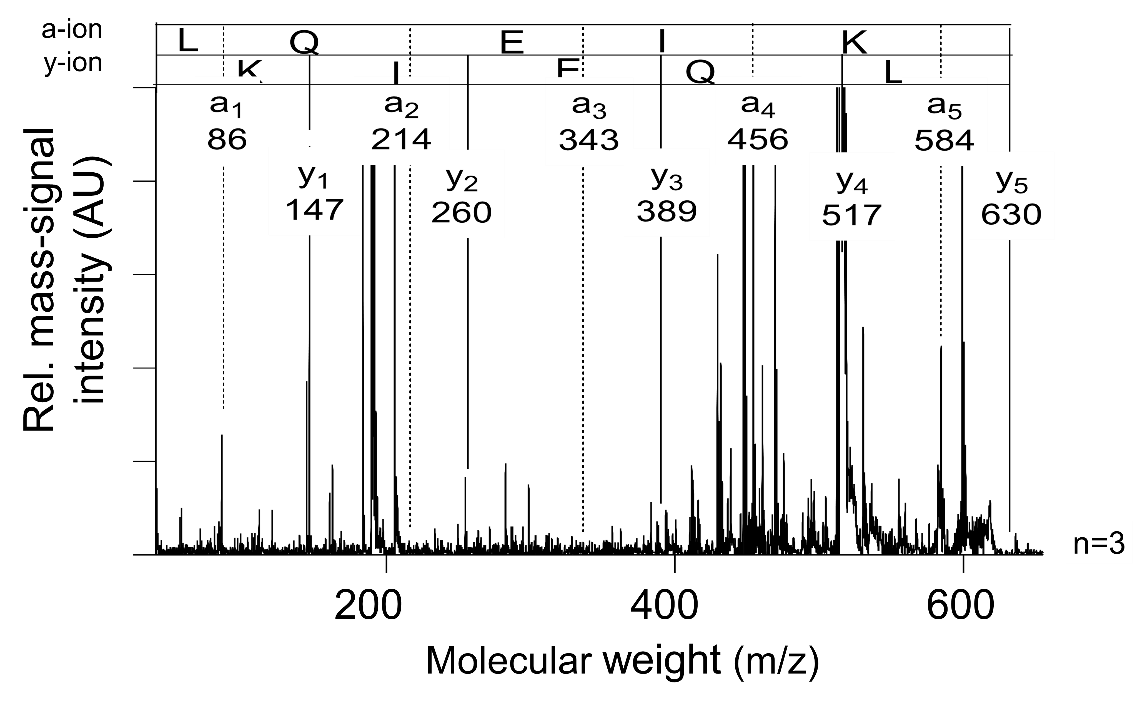


**Figure S3**: Characteristic fragment mass spectrum of chrysin-treated GGCX showing an oxidation at the molecular mass 658da (LQ*EIK);


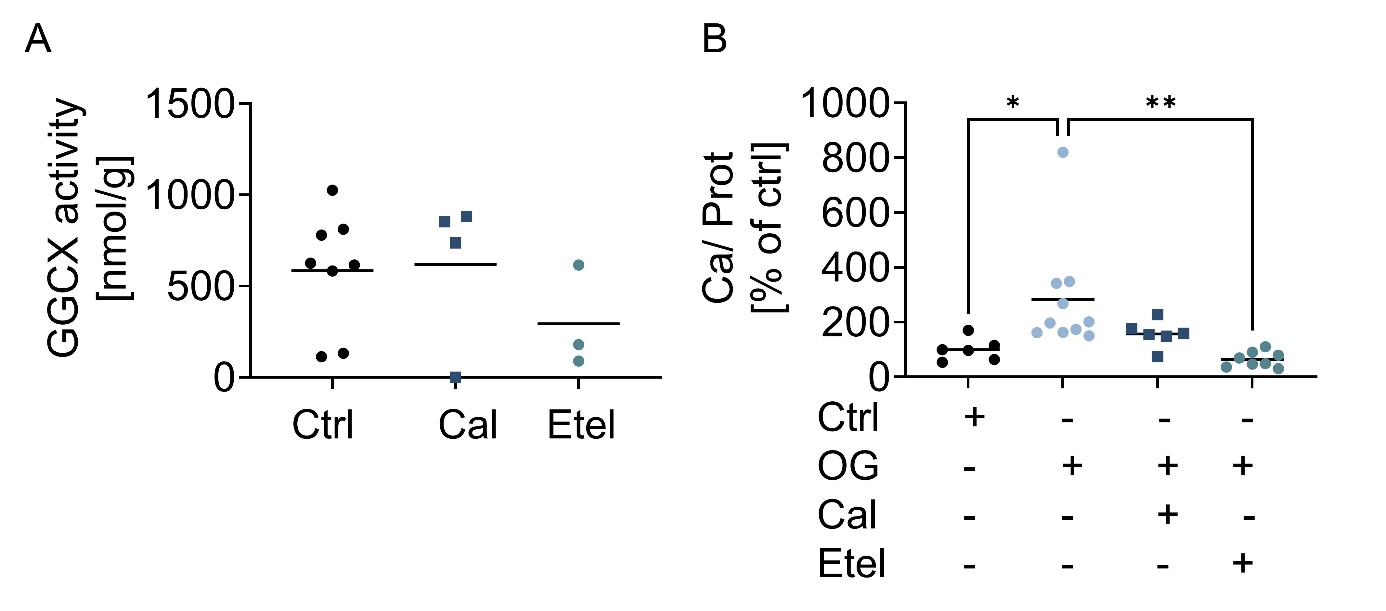


**Figure S4**: GGCX activity and calcium deposition after treatment with calcimimetica, which were suggested for potential GGCX binding via virtual compound screening; (A) GGCX activity in healthy rat liver microsomes after incubation with calindol (Cal), or etelcalcetide (Etel) to vehicle control (Ctrl); (B) calcium deposition in VSMCs after incubation with calindol or etelcalcetide, both in combination with osteogenic medium (OG), compared to normal medium control (ctrl) and to osteogenic medium alone
